# Retirement or no Retirement? The Decision’s Effects on Cognitive Functioning, Well-Being, and Quality of Life

**DOI:** 10.3390/bs10100151

**Published:** 2020-10-01

**Authors:** Carmen María Sarabia-Cobo, Victoria Pérez, Carmen Hermosilla, Pablo de Lorena

**Affiliations:** 1 IDIVAL, Universidad de Cantabria, 39005 Santander, Cantabria, Spain; 2 Geriatric Nursing Research Group, CR Santa Lucía, 40205 Madrid, Spain; vperrez.had@gmail.com (V.P.); kshavelock@gmail.com (C.H.); pdelorena.had@outlook.es (P.d.L.)

**Keywords:** retirement, cognitive abilities, aging, geriatric nursing, life satisfaction, mandatory retirement, quality of life

## Abstract

This study addressed the psychological effects on personal well-being and reported quality of life of staying professionally active in late adulthood, and to what extent years of professional inactivity modulates cognitive abilities. Design and Methods: We collected data on 262 elderly adults, 129 of whom were professionally active elderly adults (who voluntarily maintained their professional activity after the age of retirement) and 133 of whom were retired adults, in a set of experimental tasks to measure basic cognitive resources. The study took place during the first quarter of 2020. Results: Active elderly people performed better on cognitive tasks that assessed attention, memory, and solving abilities and also reported more satisfaction with life and their current work. Multiple linear regressions analyses revealed that years of inactivity were associated with lower cognitive performance. Mentally demanding jobs were significantly associated with memory performance, but not with attention and planning. Conclusions: An involuntary separation from professional activity in the beginning of late adulthood may cause a deeper decline of cognitive functions, poorer adaptive adjustment to the aging process, and higher dissatisfaction with the period of life the individual is going through.

## 1. Introduction

A current trend of industrialized countries is an increase in life expectancy; this trend is expected to expand to more developing countries in the near future [1]. The term “aging population” may be considered from different points of view. One of them regards it as a negative phenomenon and a burden to the wealth of growing societies, while another view recognizes that the increase in life expectancy has been one of the main advances of the last century and that today’s elderly are much healthier and feel younger than similarly aged individuals of past generations. One of the consequences of this change is that the age of retirement is, many times, dissociated from biological age [2]. Mandatory retirement, also known as enforced retirement, is the set age at which persons who hold certain jobs or offices are required by industry custom or by law to leave their employment or retire.

There is great variability in how elderly cope with retirement. For some persons, it simply means the end of employment, a new role in society, and the beginning of old age [3,4]. For others, it means a permanent liberation from stressful work [5]. However, for an increasing number of people, retirement occurs during a productive and satisfactory professional stage of life and is considered a personal loss [6,7]. 

Which factors determine each individual’s ability to cope with retirement? Recent studies have shown that health status and an active lifestyle contribute to voluntarily staying employed past normal retirement age (in Spain and in several European countries the normal retirement age is at 67 years) [8]. Another factor that is positively related to continuing working is the satisfaction reported with one’s professional activity (i.e., possibilities for development and influence at work, responsibility for others, meaningful work, and satisfaction with working time arrangements) [9,10]. In sum, individuals who reach normal retirement age and who feel physically and mentally able to work are more likely to suffer negative consequences of this forced transition, as they have to leave a pleasant professional activity that provided them with major satisfaction for a new, unknown role in society [11,12,13].

Several experimental studies have shown that a lack of activity in old age may have negative consequences on health status and on personal well-being [14,15,16,17] and, ultimately, affect basic daily life activities [18]. However, there is surprisingly little scientific knowledge about the consequences of retirement on cognitive abilities. Cognitive aging is defined by a progressive reduction in the brain’s weight and volume [19]. This loss is not homogeneous; indeed, it is greater in the frontal lobes [20]; therefore, the cognitive functions (e.g., executive functions) mediated by this brain region are the first to show age-related decline [21]. It is well known that some external factors may influence the cognitive aging process, such as physical exercise, diet, etc., and all recent studies indicate the need for further specific studies [22,23,24,25].

One of the main factors impacting retirement effects is the type of professional activity maintained through adulthood. This aspect has already been investigated by Phillipson (1987), who reported: “*it is in the retirement transition that the individual calls upon the resources he or she has developed during the early and middle phases of the life course. In this sense the transition is not a movement from an old to a completely new life… rather it is the final resolution of the advantages and disadvantages attached to given social and class positions*.” Phillipson studied retirement transition in three different occupational groups: miners, car factory workers, and architects. The retirement transition was easiest for the architects because they could redirect their skills (painting, carpentry, and modeling) into the increased free time of the retirement period. For the automotive workers, the situation was more problematic because the gap between work and retirement was large. For miners, the retirement transition was quite different—in the traditional mining community there is also a place for retirees, so the transition was not an individual burden, but merely a collective phenomenon. For the miners, retirement meant the consolidation of an existing level of activity; for the architects, expansion and diversification of activity; and, for many automotive workers, loss of activity [26].

A second factor to consider is the amount of professional inactive time since retirement. A theoretical interpretation of the negative effects of retirement over cognitive resources is based on the “*use it or lose it*” principle, known as the *disuse* hypothesis, or the idea that there are qualitative differences in either the structure or the process of cognition across the adult years. According to this hypothesis, intensive and continuous use of basic cognitive abilities during adulthood would prevent such abilities from undergoing the usual age-related decline (for a review, see Marquié, 1998). Evidence supporting the disuse perspective consists of comparisons of age trends in people with different amounts of relevant experience. The disuse interpretation would predict less age-related decline for people who have used their abilities continuously than for people who seldom use their abilities because, in the former case, there would have been no period of disuse. Recent work in neuroscience supports this hypothesis and shows that adult brain plasticity turns out to be greater than expected [27,28,29]. One example is found in the study on taxi drivers by Maguire, Gadian, Johnsrude, Good, Ashburner, et al. (2000) [30]. This study demonstrated that taxi drivers had larger hippocampi than did controls, suggesting that the sustained role of this structure for wayfinding increased its volume. Moreover, the more experienced and older the driver, the larger the difference was relative to controls. However, although this prediction seems quite plausible, moderate to large age-related differences have frequently been found in samples of adults with extensive amounts of experience in relevant activities. For example, similar age-related declines in measures of spatial ability have been found across groups of people with widely varying amounts of experience with spatial activities, including architects (e.g., [31,32]. Parallel age relations have been found for memory for music regardless of the individual’s amount of musical experience [33]. A major limitation of this hypothesis is that affective and motivational aspects are not considered, and the maintenance of cognitive functions is attributed uniquely to experience and/or training.

A broader approach on retirement effects is offered by the hypothesis that individuals can develop personal resources that help them resist cognitive changes associated with aging [34,35,36]. According to this point of view, successful aging depends on the extent of the physiological, cognitive, motivational, and social resources available for the individual. There is continuous feedback between maintaining physiological and cognitive abilities and personal satisfaction with one’s own life or work. For instance, the effort invested in a task may be related to self-efficacy beliefs [37]. More positive self-efficacy beliefs are expected to be associated with cognitively complex, demanding jobs, and with higher memory performance [38]. 

Drawing on the literature reviewed above, the main objective of the current study is to examine the cognitive abilities and personal well-being of professionally active elderly individuals and permanently retired participants. Specifically, we evaluate retirement effects over cognitive functions primarily affected by the aging process as attention, memory, and planning (for a review, see [39]. We predict slower and less abrupt age-related cognitive decline for professionally active seniors, when compared to permanently retired participants. A second objective is to assess to what extent cognitive abilities are associated with personal well-being. Finally, the third objective is to evaluate whether years of professional inactivity or mentally demanding jobs strongly predict performance on cognitive abilities and personal well-being. 

## 2. Design and Methods

The study design was a descriptive observational study.

### 2.1. Participants

The sample was composed of a total of 262 elderly adults who volunteered to participate in this study. A sample selection was made intentionally and for convenience. The study took place during the first quarter of 2020. We tested 129 professionally active elderly individuals (who maintained their professional activity after retirement age; in Spain, the retirement age is 65 years) and 133 elderly retired individuals. The data were collected by nurses from Spain. Retired elderly people were selected from several health and social centers for old age. The overall distribution by gender and socio professional category in our sample, as presented in Table 1, was controlled (homogeneity was sought in the sample of both groups in terms of age and professional category, finding no statistically significant differences between the groups in these variables). Type of professional activity was classified in terms of mentally demanding jobs: (1) low mentally demanding (e.g., plumber); (2) medium mentally demanding (e.g., nurse); (3) high mentally demanding (e.g., professor). The recruitment criterion for all participants was a cut-off score of 27 in Folstein’s Mini-Mental State Examination, MMSE, [40]. None of the participants reported clinical depression or mood disorders, were suffering diagnosed psychiatric disorders, or had problems of alcoholism, heart diseases, cancer, or neurological disorders. Moreover, no one was taking any medication that could affect his or her cognition, such as neuroleptics, benzodiazepines, or hypnotics. 

### 2.2. Measures

A set of experimental tasks was designed to measure the efficiency of some of the basic cognitive resources considered in the literature to account for young–old differences on a wide variety of tasks (for a review, see [41]. Six tests were administered: (*i*) a selective attention task (Stroop); (*ii*) memory tasks (face recognition, immediate and delayed verbal recall, working memory task, and category fluency); and (*iii*) a solving task (Tower of Hanoi).

*Stroop test* [42]. This task assesses inhibitory processes by requiring the participants to ignore the automatic response of reading a printed word and instead name the color of ink in which the word is printed. The materials are presented on laminated cards. This test is composed of three parts. In Part I, participants first read as many words as possible in 45s; the words are *red, green*, and *blue*, written in black ink. In Part II, participants are asked to name the color of ink in which a series of *X*s are written. Finally, in Part III, they are asked to name the color of ink in which a colored word was written (e.g., the word *green* written in red ink would require the response of “red”). The interference index is calculated using the formula suggested by Golden (1978), (C-[(AxB)/(A+B)]) and issued as the performance score.

*Face recognition* (Wechsler Memory Scale-III Faces I, [43]. This task tests immediate explicit memory for faces. There are two parts to this test. In Part I, participants are shown a series of 24 photographs of faces, one at a time, and asked to remember each one. In Part II, participants are shown a second series of 48 photographs, one at a time, half of which includes photographs of faces they had already shown with instructions to remember them. Subjects are instructed to respond “yes” if the face is one that they have been instructed to remember or “no” if it is not. A maximum raw score on immediate facial memory would be 48.

*Immediate and delayed verbal recall* (Wechsler Memory Scale (WMS-III-R); [43]. WMS III logical memory Part 1 and 2 indicate immediate and delayed recall of a short paragraph, respectively, that tests explicit memory. In Part I, two short stories are presented aurally. The second story is read twice, and the participant is asked to remember both stories. In Part II, the participant has to recall the first and second story (in this order) presented in Part I. Next, they are instructed to answer a series of questions about both stories, with a short answer (i.e., yes or no). There exists an administration and score manual, with demographically adjusted norms. This is taken as a score, as well as the mean immediate memory and delayed memory.

*The 2-back* [44]. This task requires maintenance (participants have to hold the words online as they are presented), updating (participants have to constantly update the words they are holding online), and inhibition of motor responses (because a target occurs less frequently than a nontarget, participants have to inhibit the tendency to always press the nontarget button). The stimuli are 30 nouns presented one at a time at a rate of one word every 2 s. Participants observe stimuli on the computer monitor and are asked to press one of two buttons (target) whenever the current stimulus matches the stimulus that has been presented two words before it and to press another button (nontarget) whenever the stimulus is not a match to the stimulus that occurred two previously. On 30% of the trials, the item is a match; on 70% of the trials, the item is not a match. Percentage of hits, percentage of false alarms, and the corrected hit rate (percentage of hits—percentage of false alarms) are computed. 

*Category fluency* [45,46]. Participants are asked to generate as many words as possible in one minute that are members of a given category (e.g., fruits). Subjects generate words from six categories (fruits, toys, parts of the human body, animals, articles of clothing, and professional tools) and are instructed not to give the same exemplar more than once. All fluency scores are derived by summing correct utterances and excluded repetitions. In our study, no standardized measures were used because the goal is not diagnostic but comparative.

*Tower of Hanoi* [47]. The Tower of Hanoi (TOH) is a complex problem-solving task that measures the ability to maintain an appropriate problem-solving set for the attainment of a future goal. It consists of three pegs fastened to a stand and of eight circular disks, each having a hole in the center. The disks, all of different radii, are initially placed on one of the pegs, with the largest disk on the bottom and the smallest on top; no disk rests upon one smaller than itself. The task involves transferring the individual disks from one peg to another so that no disk ever rests on one smaller than itself, and, finally, to transfer the tower; i.e., all the disks in their proper order, from their original peg to one of the other pegs. For the 8-disk problem, the puzzle requires 210 or 255 transfers. No time limit was imposed. The TOH scoring includes total correct number of tasks and number of moves beyond minimal moves. Fewer movements indicate better performance on the TOH. In our study, no standardized measures were used because the goal is not diagnostic but comparative.

*Philadelphia Geriatric Morale Scale (PGMS), revised* [48]. This scale is one of the best-known instruments for measuring well-being in the elderly. The 17-item instrument consists of three different submeasures: (1) agitation, characterizing the anxiety experienced by the elderly person (e.g., *“Do little things bother you more this year?”*); (2) attitude toward one’s aging, composed of items which relate to the older person’s attitude toward the aging process he or she experiences (e.g., *“Do things keep getting worse as you get older?”*), and (3) loneliness dissatisfaction, assessing the acceptance or dissatisfaction with the amount of social interaction the elderly person is presently experiencing (e.g., *“How lonely do you feel?”*). High scores define a basic sense of satisfaction with oneself, a feeling that there is a place in the environment for oneself, and an acceptance of what cannot be changed. In order to simplify the response task for the participants of this study, a yes/no answer format was selected. Test–retest reliability ranged from 0.91 after five weeks to 0.75 after three months.

*Professional Satisfaction Questionnaire, S4/82 (PSQ)* [49]. The S4/82 questionnaire is an 82-item measure of job satisfaction that scores 6 factors: (1) satisfaction with supervision and participation in the organization (e.g., *“How satisfied do you feel with the opportunities offered by your company to realize tasks related to your expertise?”*); (2) satisfaction with physical work environment (e.g., *“How satisfied do you feel with the physical environment and space you dispose of at work?”*); (3) satisfaction with job compensations and other rewards (with exception of salary) (e.g., *“How satisfied do you feel with training opportunities offered by your company?”*); (4) inner job satisfaction (e.g., *“How satisfied do you feel with possibilities for creativity offered by your job?”*); (5) satisfaction with salary, social service, and with security (e.g., *“How satisfied do you feel with social security or other insurance and/or benefits you receive?”*); (6) satisfaction with human relations (e.g., *“How satisfied do you feel with your personal relations with your bosses?”)*. Response type was given in a seven-point Likert-type scale. High scores indicate which factors are more satisfactory and low scores indicate which ones may need to be adjusted or changed. It maintains a substantial internal consistency (alpha 0.90) and has good levels of external validity criteria.

### 2.3. Data Collection

Participants were tested individually by the same experimenter in a well-lit, quiet room. For each individual, the study was carried out in three separated sessions held on different days. Cognitive status was evaluated by MMSE. In the first session, the participants performed the Stroop task and face recognition. During the second session, participants performed TOH, 2-back, and category fluency. In the last session, immediate and delayed verbal recall (WMS-III-R) were tested and the PGMS and the PSQ were completed. All participants were informed that they could withdraw from the study at any point and for whatever reasons.

*Ethical considerations.* Information was gathered on current and past working conditions, health, and personality. All participants reported normal or corrected-to-normal vision and hearing. Oral or written informed consent was required at the beginning of the study. The participants provided all this information to the researchers. Ethics for research involving human subjects were approved by the Ethical Board of Cantabria, Spain, with the number 27/2019CTR. 

*Statistical analysis.* IBM SPSS Statistics 25 software was used for statistical analysis. A bilateral contrast and a 95% confidence level was adopted. A descriptive analysis of all the variables collected was performed for each group. Parametric assumptions (linearity, homoscedasticity, and outliers) were visually inspected, and the normality of the distribution was verified. A MANOVA analysis was carried out to see the possible differences between the variables studied, taking as an independent variable to be or not to be retired. Statistical significance was set at *p* < 0.05.

## 3. Results

In order to examine the cognitive abilities and personal well-being of professionally active elderly individuals and permanently retired elderly, we performed a mixed analysis of two (group: active, retired) * six (task: Stroop, Face Recognition, Verbal Recall, 2-back, TOH, Category Fluency) factors. The same design was used to assess personal well-being with two (group) * two (questionnaire) factors. 

### 3.1. Effects of Retirement on Cognitive Abilities and Personal Satisfaction

**Figure 1:**
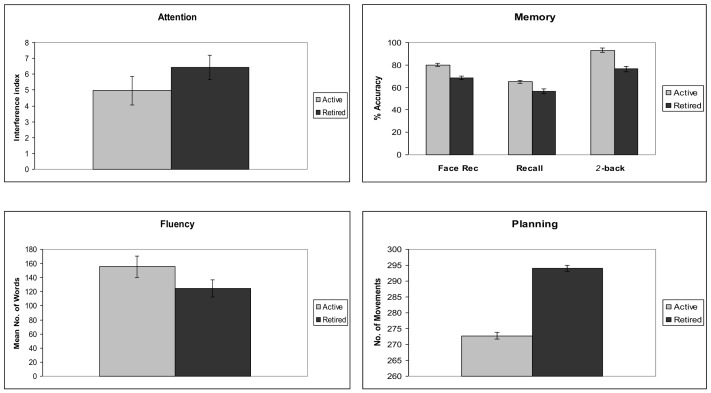
Results of active and retired participants in the different cognitive tasks.

The MANOVA revealed that performance on the Stroop task showed a significant group effect (F(1,78) = 14.25, Mean Square Error (MSE) = 2.76, *p* < 0.001), revealing less interference in active versus retired elderly (MSE−4.85 and −6.22, respectively). Active elderly also showed better memory in all tasks. On Face Recognition, active elderly correctly recognized more stimuli than retired elderly, (F(1,78) = 39.26, MSE = 5.21, *p* < 0.001) (ms 20.54 and 16.57 over 25, respectively). On Immediate and Delayed Verbal Recall, the main effect (F(1,78) = 15.84, MSE = 78.84, *p* < 0.001) showed that active elderly better recalled the two stories than the retired group (ms 63.85 and 58.67, respectively). The 2-back condition of the N-back task (F(1,78) = 25.56, MSE = 0.574, *p* < 0.001) was also significant. Accuracy (hits minus false alarms) of the active group was higher than that of the retired participants (ms 4.79 and 3.89 over 5, respectively). The main effect of Category Fluency (F(1,78) = 19.92, MSE = 985.50, *p* < 0.001) revealed a higher number of words generated by active elderly (M 159.52) versus retired elderly (M 129.86). Analysis of TOH also showed a different performance of active and retired elderly (F(1,78) = 5.95, MSE = 47.82, *p* < 0.05). Active elderly completed the task with fewer movements (M 279.66) than retired elderly (M 299). Figure 1 illustrates the results from all cognitive tasks.

A further MANOVA computed on scores from questionnaires indicated a group effect on the PGMS (F(1,78) = 61.06, MSE = 2.68, *p* < 0.001), revealing that active elderly reported more satisfaction with oneself (m 16.71) than retired participants (m 14.16). A significant effect was also found on the PSQ (F(1,78) = 30.77, MSE = 8365.85, *p* < 0.001), with active participants indicating more satisfaction towards their professional activity (m 433.19) than retired participants towards their past activity (m 355.96).

### 3.2. Correlates of Quality of Life 

To evaluate the second objective, to what extent cognitive abilities were associated to personal well-being, we computed Pearson correlations between results from cognitive tasks and scores from PGMS. PGMS scores positively correlated with performance on Stroop (r = 0.46, *p* < 0.05), Face Recognition (r = 0.66, *p* < 0.001), Verbal Recall (r = 0.71, *p* < 0.001), 2-back (r = 0.72, *p* < 0.001), Category Fluency (r = 0.55, *p* < 0.001), and negatively with TOH (r = −0.58, *p* < 0.001). A higher feeling of satisfaction was revealed by participants with better attention, memory, and planning abilities. The latter indicates that seniors who better performed TOH (i.e., fewer movements) reported higher ratings of satisfaction with life.

### 3.3. Predictive Variables of Cognitive Abilities and Personal Well-Being

In order to control for possible confounders, we then performed multiple linear regressions with years of inactivity and mentally demanding job as predictors of cognitive abilities and personal well-being with life and work. Years of inactivity was associated to lower performances as shown by negative β coefficients, with the exception of TOH. Except in one case (for TOH), being professionally active in old age was significantly associated with better cognitive abilities. Mentally demanding job was significantly associated to the four memory variables (i.e., Face Recognition, Recall, 2-back, Category Fluency), but not to attention (Stroop) and planning (TOH). Results are shown in Table 2. 

## 4. Discussion

The main objective of the current study was to examine cognitive abilities and personal well-being of professionally active elderly individuals and permanently retired elderly participants. We predicted slower and less abrupt aging effects for professionally active elderly individuals when compared to retired participants. The results showed that professionally active elderly people present better performance on the entire set of cognitive tasks assessing attention, memory, and solving abilities. Moreover, active participants manifested more personal satisfaction with life and their current work. These data support other studies that have shown that elderly people who do not engage in some activities—as, for instance, those related to work—lose the corresponding abilities [8,12,24]. For instance, Schooler, Mulatu, and Oates (1999) already showed that elderly individuals engaged in complex work into late adulthood revealed increased intellectual functioning. Similarly, Bosma, van Boxtel, Ponds, Houx, Burdorf, et al. (2003) provided longitudinal data from the Maastricht Aging Study (MAAS), revealing that elderly people with high mental work demands showed less cognitive impairment compared to their counterparts with few work demands. In more recent studies, such findings show that continued mental stimulation in late adulthood may protect against cognitive impairment [50]. Further evidence of this notion is provided by the results of the current study indicating that the effects of retirement negatively affect the maintenance of cognitive functions [14,24]. Moreover, these losses seem not to be limited to specific abilities, but rather extend to more general functions, as revealed by attention, memory, and planning tasks [51]. In light of these findings, it can be suggested that keeping mentally active in elderly people while continuing a satisfactory job, may decelerate the general functional losses of the cognitive aging process [52].

With respect to questionnaires, the results have shown that maintaining professional activity in the beginning of elderly life positively influences individuals’ self-perception, as well as satisfaction with life. It seems more adaptive, then, at least in some professions, to follow with usual activities, causing a slower and less abrupt transition from an active professional life to retirement. This aspect was highlighted by van Solinge & Henkens’ study (2008), showing that mandatory retirement had a negative effect on satisfaction with transition and affected the elderly’s quality of life. These authors underlined the importance of control over the individual’s environment to maintain well-being in late adulthood and suggested that, to understand satisfaction with retirement, one must consider how the elderly worker retired (involuntary vs. voluntary), as well as from which job they retired. Retirement experiences may vary according to the elderly’s job characteristics—the end of a physically demanding job may have a positive effect on life satisfaction, whereas, as our data reveal, the end of a stimulating job produces personal discontent and a low perception of quality of life [9,11].

A second objective was to assess to what extent cognitive abilities were associated to personal well-being. Our study shows that a probable decrease in cognitive functions (i.e., more interference, poorer memory, less ability to discover and apply logical rules) in retired elderly people seems to be directly associated with a lower satisfaction with oneself, a feeling that there is not a place in the environment for oneself and more dissatisfaction with the amount of social interaction one is presently experiencing (PGMS). These findings suggest that a radical rupture with professional activity in late adulthood may have several consequences: (a) a deeper decline of cognitive functions; (b) a poorer adaptive adjustment to the aging process; and (c) a higher dissatisfaction with the period of life an individual is going through. These findings have been found in other studies although less specifically [22,23,34].

Finally, the third objective was to evaluate whether years of inactivity (retirement) or mentally demanding jobs predict performance on cognitive abilities and personal well-being. The results seem to be associated in that years of inactivity negatively seem to affect performance in cognitive tasks, with the exception of planning performance (TOH). The ability to operate strategically seems to be independent of years of inactivity. This ability is probably more related to an individual’s capacity to functionally manage and organize their daily life activities. Moreover, more years of inactivity from retirement cause a worse perception of life satisfaction (PGMS) and less satisfaction with past work (PSQ) [53,54]. More or less satisfaction with work seems to depend on high and low mentally demanding jobs, respectively, whereas the type of professional activity does not influence the perception of life satisfaction. These last data indicate that, overall, the amount of years of inactivity increase cognitive decline in late adulthood. This decline would affect all cognitive abilities in the same degree. Furthermore, years of inactivity seem to be associated with the degree of satisfaction of individuals in old age [22,36,50]. That is, as years go by, retirees report a lower perception of worthwhile living. 

The second variable considered as a predictor of cognitive decline was the type of activity maintained during adulthood. Our results show that this variable would be a good predictor of cognitive decline in tasks that are not associated with an individual’s knowledge, such as attention and planning. Other studies have found findings in this regard but not as specifically as our study [4,12]. In memory tasks, participants with low mentally demanding jobs are more vulnerable to developing cognitive decline. However, mentally demanding jobs are not associated with affective variables [55]. Overall life satisfaction does not depend on the type of professional activity [5]. On the contrary, the latter explains the degree of satisfaction with past or current work. Individuals with highly mentally demanding jobs report more satisfaction with the maintenance of professional activity even in late adulthood, a period of life that is socially related to retirement [56,57].

The results of this research are novel because as far as we know, it has studied the possible relationship between good quality of life, adequate cognitive functions, and staying active at work. Other studies have exploited these areas, but not jointly. Studying both the cognitive and the emotional impact of stopping working provides us with interesting suggestions for tackling this problem in a global way.

## 5. Conclusions

In conclusion, this study reveals a further way to understand the effects of retirement on the cognitive abilities of the elderly and the role of such effects on the individuals’ affective factors. Beside the political or social aspects of retirement, it would be convenient to consider that it is one of the major changes in life, and the step into this new stage should be voluntarily decided by each individual in order to avoid personal negative effects. Moreover, because of increased longevity and improved health status, individuals at retirement age could partially maintain their job to continue with the activities that provide them with satisfaction. This point should make us reflect on which is the best moment to retire, especially in more developed countries where life expectancy has considerably increased and fitness to work is not considered at the age of legal retirement. The assessment of functional capacity of the elderly to continue working should include a good understanding of the nature of the work and consider possibilities for work accommodations. Retirement can mark the beginning of a new life where individuals continue to exercise their abilities, to a certain degree, or it can be seen as the undesired end of a satisfactory activity. The results of this study suggest the convenience for individuals to freely choose whether, and to what extent, to retire from professional activity. This would imply major benefits for old persons, such as less cognitive decline and a better perception of life. Together, these aspects are basic factors of successful aging.

## Figures and Tables

**Table 1:** Participant characteristics.

Group	*n (M, F)*	Age (S.D.)	Years of Inactivity	Mentally Demanding Job (S.D.) *	MMSE ** (S.D.)
Active	129 (40:36)	68:98 (2.76)	0	2.32 (0.79)	30 (0.69)
Retired	133 (45:41)	69 (2.95)	5.84 (2:88)	2.01 (0.92)	29.02 (0.44)

* Mentally-demanding job was coded as: (1) low, (2) medium, (3) high mentally-demanding. ** Mini Mental State Examination (Folstein, Folstein & McHugh, 1975).

**Table 2:** Regression models for years of inactivity and professional activity on different measures of cognitive abilities and personal well-being.

	Years of Inactivity			Professional Activity		
***Cognitive Abilities***	β	*p*	*R^2^*	β	*p*	*R^2^*
Stroop	−23	0.000	0.19	0.15	0.58	0.00
Face Recognition	−46	0.000	0.36	1.51	0.000	0.24
Recall	−1.15	0.000	0.18	7.32	0.000	0.45
TOH	2.31	0.080	0.06	−1.31	0.080	0.00
Category Fluency	−3.86	0.000	0.19	30.02	0.000	0.60
2-back	−0.15	0.000	0.26	0.51	0.000	0.26
***Personal Satisfaction***						
PGMS	−0.39	0.000	0.46	0.79	0.005	0.09
PSQ	−16.19	0.000	0.26	67.31	0.000	0.29
